# Mental health literacy of Internet gaming disorder and problematic smartphone use among Korean teenagers

**DOI:** 10.1371/journal.pone.0270988

**Published:** 2022-07-21

**Authors:** Mina Jeon, Mi-Sun Lee, Ji-Young Yoon, Soo-Young Bhang

**Affiliations:** 1 Department of Psychology and Human Development, Institute of Education, University College London, London, United Kingdom; 2 Department of Preventive Medicine, College of Medicine, The Catholic University of Korea, Seoul, Korea; 3 Center for School Mental Health, Eulji University, Seoul, Korea; 4 Department of Social Welfare, Ewha Womans University, Seoul, Korea; 5 Department of Psychiatry, Eulji University School of Medicine, Nowon Eulji University Hospital, Seoul, Korea; 6 Nowon Community Addiction Management Center, Seoul, Korea; PLOS, UNITED KINGDOM

## Abstract

The last few decades have seen an increased interest in ‘Mental Health Literacy (MHL)’ which addresses knowledge and beliefs relating to mental health problems and is likely to hinder treatment-seeking behaviors. Although MHL research to date has explored various mental disorders, far too little attention has been paid to Internet Gaming Disorder (IGD) and Problematic Smartphone use (PSU). The objective of this study is to develop an MHL questionnaire for IGD and PSU by adopting the MHL approach in the previous literature and examine MHL of IGD and PSU in Korean teenagers by focusing on their ability to recognize addictions, and perceptions and help-seeking attitudes towards a person with addictions. The current study also aimed to compare Korean teenagers’ MHL depending on low- and high-risk of IGD and PSU. A total of 169 teenagers aged 10 and 16 years were recruited from schools and children’s centers in Seoul and completed the newly developed MHL questionnaire and screening questionnaires for IGD and PSU. The MHL questionnaire for IGD and PSU was designed as a vignette-based questionnaire that depicted behavioral characteristics of a severe problem with Internet and smartphone usage. Korean teenagers had a poor ability to recognize IGD and PSU and were more prone to seek parental help than professional help. The current study also showed that teenagers had better recognition of IGD than PSU and perceived that a person with IGD has a more difficult life than a person with PSU. Furthermore, a low-risk or high-risk group of IGD or PSU showed minimal differences in MHL. The current study provided empirical evidence to support a need to develop educational programs to improve Korean teenagers’ MHL for both IGD and PSU.

## Introduction

In developing health interventions to reduce risk behaviors, existing research recognizes the critical role of providing information tailored to individuals’ unique needs [1]. With growing concerns about mental health problems worldwide, it is crucial to develop mental health interventions tailored to individuals or groups to promote their early and appropriate treatment-seeking behaviors. The last few decades have seen an increased interest in ‘Mental Health Literacy’ which addresses knowledge and beliefs relating to mental health problems and is likely to hinder treatment-seeking behaviors. Jorm et al. (1997) developed the term ‘Mental Health Literacy’ (MHL) based on the concept of health literacy [2, 3]. MHL was defined as a person’s knowledge and beliefs about mental disorders that aid their ability to recognize specific mental health problems and manage and prevent their mental health problems [2]. According to Jorm et al. (1997), MHL consists of the following six components: 1) the ability to recognize specific disorders, 2) the knowledge of how to seek mental health information, 3) the knowledge about risk factors and causes of mental disorders, 4) the knowledge of self-treatment, 5) the knowledge about the availability of professional help, 6) the attitudes that promote the recognition of mental disorders and seeking appropriate help [2].

Jorm et al. (1997) employed a vignette-based method to examine a person’s ability to recognize the presence of depression and schizophrenia and utilized a rating method to investigate a person’s perception on the effectiveness of various treatments for depression and schizophrenia [2]. Since then, several researchers adopted Jorm et al.’s approach to assessing a person’s MHL for other mental disorders such as eating disorders, obsessive-compulsive disorders, and post-traumatic stress disorders [4–6]. Although the earlier evidence suggests that the general public has poor MHL [2, 5–8], some recent studies investigated changes in mental health literacy and found improvements in the ability to recognize symptoms of mental health disorders [9, 10]. Extensive research has also shown that improving MHL is beneficial for appropriate help-seeking and treatment choices [11, 12]. Given the importance of improving MHL, many researchers put efforts to develop various methods to improve MHL such as community awareness campaigns [13, 14], school-based interventions [15], and mental health first aid [16].

Although MHL research to date has explored a number of mental disorders, far too little attention has been paid to behavioral addictions. There has been only one study that investigated Chinese adolescents’ MHL for Problematic Internet Use (PIU) [17] by developing a vignette-based question that presented a young person with symptoms of PIU and other questions covering an intention to seek help, perceived barriers, and exposure to the disorder. The results demonstrated that over 50% of participants correctly recognized the presence of PIU in the vignette and intended to seek help. The most preferred methods of seeking help were from friends in school, followed by a mother and a father. These findings represent that Chinese adolescents have a moderate ability to recognize PIU but preferred to seek unprofessional help [17]. Although this study has an implication of investigating the MHL of PIU for the first time, the study has investigated community samples only. However, a previous study on mental health literacy of eating disorders has found that women with high risk or symptoms had different attitudes to seek help and perception of the eating disorders across groups [18]. Therefore, there remains a lack of evidence on whether participants may have different MHL of PIU depending on the risk profiles of PIU. Furthermore, none of the studies examined MHL for Problematic Smartphone Use (PSU) to the best of the authors’ knowledge. However, with the rapid development of technology over the past few decades, an increasing number of people utilizes not only the Internet but also smartphone. Although there is no doubt that the use of the Internet and smartphone brings convenience and entertainment to our society, uncontrollable, excessive use, and over-dependency on them may lead to IGD and PSU. Given the concerns on problematic use of the Internet and smartphone, IGD was included in the 5^th^ edition of the Diagnostic and Statistical Manual of Mental Disorders (DSM-5) as a condition for further study [19]. Similarly, the World Health Organization (2019) has also decided to include gaming disorders (both online and offline) in the eleventh edition of the International Statistical Classification of Diseases and Related Health Problems (ICD-11) [20]. Although PSU is still a controversial topic requiring a further investigation to be officially recognized as a disorder, previous literature stressed that PSU has several characteristics similar to substance-related disorders in the DSM-5 such as compulsive behavior, functional impairment, withdrawal, and tolerance [21]. Therefore, previous studies developed and implemented measurements for PSU, often based on the DSM-5 criteria for IGD [19] or by adapting standardized internet addiction tests [22–24].

Despite the necessity of further investigation on the diagnostic criteria of IGD and PSU, many studies investigated the prevalence of IGD and PSU among teenagers in various countries [25–31], and comparably high prevalence of IGD and PSU were found in South Korean adolescents [26, 28]. Previous studies also investigated the impact of IGD and PSU on teenagers’ daily functioning and found adverse effects on teenagers’ physical and psychological health, social functioning, and cognitive functioning [25, 32–35]. Therefore, adolescents’ IGD and PSU have become critical issues that have to be resolved. However, most studies have focused on investigating how IGD and PSU affect teenagers’ daily functioning, and little is known about how adolescents recognize, perceive, and manage a problem of IGD and PSU. However, teenagers’ MHL for IGD and PSU has to be studied to ensure that teenagers have appropriate help-seeking attitudes and treatment choices. Therefore, the current study aimed to develop a MHL questionnaire for IGD and PSU by adopting the MHL approach and examining the MHL of IGD and PSU among teenagers with low risk and high risk of IGD or PSU. The current study focused on teenager’s 1) ability to recognize IGD and PSU, 2) their perception or attitudes towards a person with IGD and PSU and 3) their help-seeking attitudes towards a person with IGD and PSU including self-treatment and professional help. Furthermore, the current study compared teenagers’ MHL depending on the low risk and high risk of IGD and PSU.

## Materials and methods

### Participants

From March to October 2017, all the schools and community child care centers in one district, Nowon-gu in Seoul, South Korea were provided with an official letter that informed the purpose, components, and procedure of the 2017 smart digital media survey project conducted by Nowon Community Addiction Management Center [36]. From 23 schools and 11 community child care centers in Nowon-gu, 3,937 participants were recruited. Among these participants, researchers provided information sheets and consent forms for this study to participants who satisfied at least one of the criteria: 1) IGUESS score ≥ 10; 2) SAS-SV score ≥ 23; 3) hours of Internet use per day ≥ 3hr (for participants in elementary schools) or ≥ 4hr (for participants in middle or high schools); and 4) experience of Internet gambling or online payments ≥ 80 dollars per annum. A total of 180 consent forms were obtained and 169 teenagers from 9 schools and 10 community childcare centers participated in the study with a response rate of 93.89%. Of the 169 teenagers, the results of 7 participants were excluded from the analysis because they did not respond to all items related to the MHL questionnaire. Participants who missed a few items were still included since this study conducted a separate analysis for each item. As a result, the final sample consisted of 162 teenagers (M_age_ = 12.4, SD = 1.19). The demographic characteristics and screening outcomes of the IGD and PSU were presented in Table 1. Ninety-one participants (53.8%) were male, and 78 participants (46.2%) were female. Given the consent from the parents of teenagers, teenagers completed a Mental Health Literacy (MHL) questionnaire when they were having an educational program regarding addiction disorders at their school. Teenagers also completed screening questionnaires for IGD and PSU such as Internet Game Use-Elicited Symptom Screen (IGUESS) [37] and Smartphone Addiction Proneness Scale–Short Version (SAS-SV) [38]. Teenagers who scored greater than or equal to 10 on IGUESS and greater than or equal to 23 on SAS-SV were grouped into risk groups of IGD and PSU, respectively. The rest of the participants were classified as low-risk groups of IGD and PSU. Twenty-two participants (13%) fell into a risk group of IGD while 30 participants (17.8%) fell into a risk group of PSU.

**Table 1:** Demographic characteristics of the participants.

		N (%)
**Age (years)**	10	1 (0.6)
	11	27 (16.7)
	12	87 (53.7)
	13	20 (12.3)
	14	10 (6.2)
	15	16 (9.9)
	16	1 (0.6)
**Gender**	Male	85 (52.5)
	Female	77 (47.5)
**IGUESS**	1~9	141 (87.0)
	≤10	21 (13.0)
**SAS-SV** ^a^	≥22	132 (82.0)
	≤ 23	29 (18.0)

IGUESS, Internet Game Use-Elicited Symptom Screen [37]; SAS-SV, Smartphone Addiction Proneness Scale–Short Version [38].

^a^ n = 161.

### Measures

#### Mental health literacy questionnaire

A mental health literacy questionnaire for IGD and PSU was developed based on the concept of MHL questionnaire in the previous literature [5] that examined the Korean public’s mental health literacy for nine mental disorders. The MHL questionnaire used in Jeon and Furnham’s study [5] included five components of MHL except for the knowledge about risk factors and causes of mental disorders and organized them into three sections such as 1) the ability to recognize specific disorders, 2) the attitudes to characters that promote the recognition of mental disorders and seeking appropriate help, and 3) the knowledge and attitudes towards help-seeking including self-treatment and professional help [5]. The current study only revised vignettes used in Jeon and Furnham’s study [5] and developed two vignettes that describe characters with IGD and PSU respectively. The content validity of the developed vignettes on IGD and PSU was examined by eight professionals who have rich experience with teenagers with addiction problems (in the field of psychiatrist, psychology, mental health and social welfare, and rehabilitation therapy). They assessed the developed mental health literacy questionnaire with the aim of evaluating the correct description of the symptoms of young children with IGD and PSU, the completion time of the questionnaire, and the use of correct grammar and appropriate words. Based on the evaluation of the professionals, necessary amendments were made by adding examples of social media platforms or online games that are child-friendly for describing the symptoms of children with IGD or PSU, correcting spelling and grammar errors, and revising words to ensure that children could easily understand the questions.

*The ability to recognize IGD and PSU*. The questionnaire consisted of two vignettes depicting cases with IGD and PSU. The IGD vignette presented a young child with the characteristics of IGD described in the DSM-5 and reflected typical problems of young children with IGD based on the clinical experiences of addiction professionals. The PSU vignette was also developed by reflecting the core characteristics of IGD described in the DSM-5 but depicted a young child with overuse of a smartphone. The developed vignette was checked and confirmed by eight addiction professionals. An open-ended question “What, if any, do you think is wrong with the young person?” was derived from previous literature [2, 5] and was asked for each vignette to assess teenagers’ recognition of the IGD and PSU.

*Perception and attitudes towards a person with IGD and PSU*. After the open-ended question, six rating questions on a Likert scale of 1–7 (1 = not at all, 7 = extremely) were presented to examine participant’s perceptions of each vignette case’s level of distress, difficulty in treating their problems, happiness, successful academic life and satisfaction with a friendship. Participants’ empathy towards each character was also examined to assess participants’ attitudes towards IGD and PSU cases. Reliability analysis was conducted by reversely coding four questions that examine participants’ perceptions of each vignette case’s level of difficulties in a positive way (e.g., questions asking about participants’ empathy towards each character, and participants’ perceptions of the vignette case’s level of happiness, successful academic life, and satisfaction with a friendship). Cronbach’s alpha and McDonald’s omega for the six rating questions related to the IGD vignette were .578 and .584, respectively. For the six rating questions related to the PSU vignette, Cronbach’s alpha and McDonald’s omega were .582 and .636. The removal of the question asking for participants’ empathy towards IGD and PSU cases increased the Cronbach’s alpha to .623 and .723, respectively.

*Help-seeking attitudes towards a person with IGD and PSU*. Following the questions mentioned above, participants were asked to rate the likelihood of suggesting various help options for the person depicted in each vignette on a Likert scale of 1–7 (1 = not very likely, 7 = very likely). Help options can be categorized into general help, self-help, and professional help. In total, twelve help options were included, namely general help, coping alone, a teacher, a friend, a parent, other family members, school counselors, doctors, psychologists, psychiatrists, books, and the Internet. Reliability analysis was conducted on twelve help options by reversely coding one of the help options that ask participants’ likelihood of suggesting coping alone. Both Cronbach’s alpha and McDonald’s omega showed good reliability of these questions for IGD case (α = 0.83, ω = 0.84) as well as PSU case (α = 0.85, ω = 0.86).

#### Internet Game Use-Elicited Symptom Screen (IGUESS)

Internet Game Use-Elicited Symptom Screen (IGUESS) is a self-reported questionnaire developed to screen the IGD based on the diagnostic criteria of IGD in the DSM-5 [37]. Participants were asked to respond to each question on a Likert scale of 0–3 (0 = never, 3 = always) by thinking about their use of the Internet and game in the past 12 months. A clinical cut-off score is 10. Jo et al. (2017) investigated 10–19 years old Korean adolescents to check the validity and reliability of IGUESS and found a high correlation coefficient with Young’s Internet Addiction Test (r = 0.902) [37]. Also, the sensitivity and specificity of IGUESS were 87.0, and 86.7%, respectively, and Cronbach’s alpha was 0.94. The reliability of IGUESS was additionally examined in this study and found a good level of reliability of IGUESS (α = 0.82, ω = 0.83).

#### Smartphone Addiction Proneness Scale–Short Version (SAS-SV)

Smartphone Addiction Proneness Scale–Short Version (SAS-SV) developed by Kwon, Kim, Cho and Yang (2013) was utilized to measure a smartphone addiction among teenagers [38]. There are two different types of SAS-SV; one for young children and the other for adolescents and adults. The SAS-SV for adolescents consists of 10 items on a Likert scale of 0–3 (0 = not at all, 3 = strongly agree). Given the maximum score of 40, the clinical cut-off score for a high-risk adolescent is 31, and the clinical cut-off score for a potential risk adolescent is between 23 and 30. Reliability analysis was conducted for SAS-SV and found a very good level of reliability of SAS-SV in this study (α = 0.91, ω = 0.91).

### Statistical analysis

IBM SPSS Statistics Version 24.0 for Windows was used for all statistical analyses. P-values < 0.05 were considered to indicate statistical significance. *All 162 participants completed the questionnaires*, *except one participant who did not complete the SAS-SV questionnaire*. *Furthermore*, *some participants missed answering a few questions from the MHL questionnaire (N = 17)*. *Despite these missing values*, *the participants were included in the analysis and treated as missing values because their responses to other questions provided valuable information*. Participants’ ability to recognize the IGD and PSU was compared using a Chi-squared test. Wilcoxon signed ranks test was performed to see if participants’ ratings for the perception and help recommendation for IGD and PSU cases were significantly different from each other. Participants’ MHL for IGD and PSU was compared depending on the risk of IGD and PSU using a Chi-squared test and Mann-Whitney U test.

### Ethics

The study procedures were carried out in accordance with the Declaration of Helsinki. All procedures were approved by the Eulji University Hospital’s Institutional Review Board (IRB No. EMCS 2018-06-014).

## Results

### Recognition and perceived perception of the person with IGD and PSU

Participants’ responses to open-ended questions were analyzed using content analysis. Responses were categorized into ‘correct’ or ‘incorrect’ to determine how many participants correctly identified the IGD and PSU. Since there hasn’t been a consensus on the use of terminology to describe problematic internet use in the previous literature [39], a number of different labels for IGD were accepted. Responses were considered ‘correct’ labels for the IGD if participants provided the correct term or other terms like Internet game addiction, Internet addiction, and game addiction. For the recognition of PSU, smartphone addiction and SNS addiction were considered correct labels as the vignette depicted a person addicted to a smartphone, mainly due to social media use. Participants’ responses to open-ended questions were reanalyzed to see if participants recognize that a depicted person has an addiction disorder. Responses were considered to be correct if participants recognized that the depicted person is experiencing an addiction disorder. All other responses were considered to be ‘incorrect’. Interrater reliability analysis was carried out to ensure the consistency of coding between raters. As a result, the interrater reliability was significant for both strict and looser criteria for both disorders (All Kappa > 0.874, p < .001).

Table 2 shows the participants’ recognition rate of the IGD and PSU in each vignette. Participants were more likely to recognize the correct labels of IGD and identify addiction disorders in the IGD vignette compared to the PSU vignette (both p < .001).

**Table 2:** Mean of the recognitions and ratings of the character adjustment to living with Internet and smartphone addiction.

		Internet (n = 156)	Smartphone (n = 155)	*X* ^*2*^	*p* ^*c*^
**Recognition**	Correct label (%)	25.6	12.9	20.9	< .001**
	Addiction disorder (%)	29.5	22.6	22.8	< .001**
		**(n = 162)**	**(n = 160)**	*z*	*p* ^*c*^
**Perception**	Feeling difficulty	5.53 (1.79)	5.39 (1.56)	-1.88	.061
	Treatment difficulty	6.09 (1.48)	5.90 (1.31)^b^	-2.51	.012*
	Empathy	3.82 (1.96)	3.89 (2.07)	-.64	.522
	Happiness	3.53 (1.93)	3.39 (1.76)	-1.05	.294
	Successful Academic life	1.71 (1.21)	2.54 (1.59)	-6.36	< .001**
	Satisfied friendship	2.30 (1.53)^a^	2.83 (1.65)	-3.89	< .001**

Likert scale of 1–7 (1 = not at all agree, 7 = extremely agree).

^a^ n = 161.

^b^ n = 159

^c^ * p < .05

** p < .01

Participants’ mean ratings and standard deviation (SD) for their perceptions on the difficulty of living with IGD and PSU were shown in Table 2. In general, participants believed that cases with either IGD or PSU are feeling difficult to live with the problem (all > M = 5.39), difficult to be treated (all > M = 5.90), less happy (all < M = 3.5*3*), having less successful academic life (all < M = 2.54) and having less satisfied friendship (all < M = 2.8*3*), but expressed less empathy towards them (all < M = 3.89). A Wilcoxon signed ranks test was performed to see if participants’ ratings for IGD and PSU were significantly different from each other and found significant differences in ‘treatment difficulty’ (p = .012), ‘successful academic life’ (p < .001) and ‘satisfied friendship’ (p < .001). Participants perceived that a case with IGD is significantly more difficult to be treated and has a significantly less successful academic life and less satisfying friendship.

### Likelihood of suggesting help for the person with IGD and PSU

Table 3 shows the mean ratings and SD for the likelihood of suggesting help in general and the likelihood of suggesting various help options for cases with IGD or PSU. For both cases, participants recommended help from parents the most (all > M = 5.86) and the Internet search the least (all < M = 3.61). Wilcoxon signed ranks test was performed to see if participants’ ratings for the help recommendation for IGD and PSU were significantly different from each other. Significant differences between help recommendation for IGD and PSU were found in suggesting help from parents (p < .001), addiction counsellor (p = .04) and friends (p = .009). Compared to the help recommendation for the case with PSU, participants rated help from parents and addiction counselors as significantly more helpful for the case with IGD, whereas rated help from friends as less helpful for the case with IGD.

**Table 3:** Ratings for the likelihood of suggesting help for a person with IGD and PSU.

	Internet (n = 161)	Smartphone (n = 159)	*Z*	*p* ^*d*^
General help	5.34 (1.90)	5.18 (1.97)	-1.34	.180
Should cope alone	4.65 (2.16)	4.67 (2.18)^b^	-.43	.667
School teacher	5.26 (1.78)^a^	5.32 (1.70)	-.08	.934
Friends	4.93 (1.85) ^a^	5.20 (1.93)	-2.61	.009**
Parents	**6.19 (1.40)** ^a^	**5.86 (1.62)**	-3.77	< .001**
Relatives	5.26 (1.78) ^a^	5.30 (1.77)	-.29	.773
School counselor	5.74 (1.62)	5.71 (1.67)^c^	-.40	.686
Psychologist	5.55 (1.73) ^a^	5.50 (1.75) ^c^	-1.16	.245
Psychiatrist	5.01 (2.01)	4.99 (1.99) ^b^	-.82	.412
Addiction counselor	5.85 (1.65)	5.65 (1.78)	-2.05	.040*
Call or online counseling	5.17 (1.93)	5.11 (1.96)	-.84	.401
Books	4.66 (2.07)	4.78 (2.06) ^b^	-.53	.599
Internet search	3.42 (2.11)	3.61 (2.06) ^b^	-1.12	.262

Likert scale of 1–7 (1 = not very likely, 7 = very likely).

Data in bold represents the help option that had the highest ratings and underlined data represents the help option that had the lowest ratings.

^a^ n = 160.

^b^ n = 158.

^c^ n = 157.

^d^ * p < .05

** p < .01

### Comparison of MHL for IGD depending on the low and high risk of IGD

The comparison of participants’ ability to recognize the IGD depending on low and high risk of IGD was presented in Table 4. Table 4 also shows the mean ratings and SD for two groups’ (e.g., participants with low risk and high risk of IGD) perceptions towards the case with IGD and the likelihood of suggesting various help options. The results showed no significant differences in recognition of IGD and help-seeking behavior between low-risk and high-risk groups. However, participants with a high-risk of IGD had significantly higher empathy towards the case with IGD than participants with a low-risk of IGD (p = .002). Although two groups rated the Internet search the least helpful (all M < 3.46), participants with a high-risk of IGD rated addiction counselors the most helpful (M = 6.00, SD = 1.34), while participants with a low-risk of IGD rated the parents the most helpful (M = 6.22, SD = 1.31).

**Table 4:** Comparison between participants with the low and high risk of IGD.

		Low risk of IGD (n = 136)	High risk of IGD (n = 20)	*X* ^*2*^	*p* ^*d*^
**Recognition**	Correct label (n, %)	35 (25.7%)	5 (25%)	.005	.944
	Addiction disorder (n, %)	41 (30.1%)	5 (25%)	.222	.637
		**(n = 141)**	**(n = 21)**	**U**	***p*** ^***d***^
**Feeling**	Difficulty	5.50 (1.85)	5.71 (1.35)	1473.5	.971
	Treatment	6.09 (1.49)	6.10 (1.41)	1457.5	.897
	Empathy	3.62 (1.94)	5.10 (1.58)	870.0	.002**
	Happiness	3.43 (1.92)	4.19 (1.89)	1101.5	.054
	Successful	1.66 (1.19)	2.05 (1.28)	1190.0	.093
	Friendship	2.23 (1.48)^a^	2.81 (1.83)	1218.5	.183
		**(n = 140)**	**(n = 21)**		
**Help**	General help	5.39 (1.90)	4.95 (1.88)	1244.5	.238
	Alone	4.67 (2.09)	4.52 (2.60)	1459.0	.955
	School teacher	5.24 (1.78)^b^	5.38 (1.88)	1366.5	.628
	Friends	4.99 (1.73)	4.50 (2.52)^c^	1307.5	.626
	Parents	**6.22 (1.31)** ^b^	5.95 (1.91)	1459.0	.998
	Relatives	5.35 (1.70) ^b^	4.67 (2.15)	1207.5	.188
	School Counsellor	5.72 (1.65)	5.86 (1.42)	1445.5	.896
	Psychologist	5.52 (1.75) ^b^	5.76 (1.55)	1353.5	.576
	Psychiatrist	5.11 (1.98)	4.33 (2.13)	1140.5	.090
	Addiction counselor	5.82 (1.69)	**6.00 (1.34)**	1431.0	.833
	Call or online counseling	5.19 (1.89)	5.05 (2.27)	1446.0	.901
	Books	4.76 (2.00)	4.00 (2.45)	1207.0	.179
	Internet search	3.46 (2.10)	3.14 (2.20)	1335.5	.492

Likert scale of 1–7 (1 = not very likely, 7 = very likely).

Data in bold represents the help option that had the highest ratings and underlined data represents the help option that had the lowest ratings.

^a^ n = 140.

^b^ n = 139.

^c^ n = 20.

^d^ * p < .05

** p < .01

### Comparison of MHL for PSU depending on the low and high risk of IGD

The comparison of participants’ ability to recognize the PSU depending on the low and high risk of PSU was presented in Table 5. Table 5 also shows the mean ratings and SD for two groups’ (low-risk and high-risk of PSU) perceptions of the case with PSU and the likelihood of suggesting various help options. The result showed that the high-risk group recognized the correct label of PSU significantly better than the low-risk group (p = .015). There were no significant group differences in their perceptions and help recommendations towards the case with PSU, except for the general help recommendation. Participants with low risk of PSU rated the general help significantly higher than participants with a high risk of PSU (p = .049).

**Table 5:** Comparison between participants with the low and high risk of PSU.

		Low risk of PSU (n = 129)	High risk of PSU (n = 25)	*X* ^*2*^	*P* ^*f*^
Recognition	Correct label (n, %)	13 (10.1%)	7 (28%)	5.953	.015*
	Addiction disorder (n, %)	27 (20.9%)	8 (32%)	1.461	.227
		**(n = 130)**	**(n = 29)**	***U***	***p*** ^***f***^
Feeling	Difficulty	5.45 (1.56)	5.10 (1.61)	1656.0	.292
	Treatment	5.93 (1.29)^a^	5.86 (1.30)	1768.0	.627
	Empathy	3.89 (2.01)	4.00 (2.36)	1823.0	.779
	Happiness	3.34 (1.73)	3.59 (1.96)	1705.5	.415
	School performance	2.42 (1.53)	3.07 (1.77)	1474.0	.057
	Friendship	2.72 (1.61)	3.35 (1.78)	1488.5	.070
		**(n = 131)**	**(n = 29)**		
Help	Help	5.32 (1.94)^a^	4.59 (2.04)	1447.0	.049*
	Alone	4.72 (2.19)^b^	4.56 (2.06)^c^	1641.0	.587
	School teacher	5.34 (1.68)	5.33 (1.82)^c^	1743.0	.903
	Friends	5.21 (1.90)	5.26 (2.07)^c^	1707.5	.770
	Parents	**5.86 (1.63)**	**5.89 (1.63)** ^c^	1740.5	.888
	Relatives	5.29 (1.79)	5.37 (1.74)^c^	1724.5	.833
	School Counsellor	5.70 (1.69)^b^	5.77 (1.63)^d^	1633.0	.773
	Psychologist	5.50 (1.74)	5.52 (1.83)^e^	1576.5	.757
	Psychiatrist	5.04 (1.96)	4.69 (2.19)^d^	1559.5	.488
	Addiction counsellor	5.71 (1.77)	5.41 (1.85)^c^	1567.5	.320
	Call or online counselling	5.15 (1.96)	5.04 (2.01)^c^	1702.0	.752
	Books	4.80 (2.04)	4.73 (2.20)^d^	1676.5	.898
	Internet search	3.66 (2.06)	3.27 (2.05) ^d^	1532.5	.414

Likert scale of 1–7 (1 = not very likely, 7 = very likely).

Data in bold represents the help option that had the highest ratings and underlined data represents the help option that had the lowest ratings.

^a^ n = 129.

^b^ n = 130.

^c^ n = 27.

^d^ n = 26.

^e^ n = 25.

^f^ * p < .05

** p < .01

## Discussion

The purpose of the current study was to examine Korean teenagers’ MHL for IGD and PSU, focusing on the ability to recognize IGD and PSU, a perception or attitudes towards a person with IGD and PSU, and help-seeking attitudes toward a person with IGD and PSU. Another objective of the current study was to compare teenagers’ MHL depending on the low risk and high risk of IGD and PSU.

### Recognition of IGD and PSU

Our finding showed that compared to the PSU, a higher percentage of Korean teenagers recognized the correct label of IGD and identified a case with IGD as having an addiction disorder. The ambiguity in the terminology of behavioral addictions may explain this finding that only IGD has been classified as a behavioral addiction in the current version of DSM-5 and PSU still needs further investigation [19]. Uncertainty in the terminology of PSU may result in poorer ability to recognize correct terms and addictive symptoms of PSU among Korean adolescents. Although Korean adolescents showed better recognition of IGD than the recognition of PSU, their recognition rate of IGD was lower than Chinese adolescents’ recognition rate for problematic Internet use (58%) [17]. Despite the lower recognition rate of IGD in South Korea, the previous studies found a higher prevalence rate of IGD in Korean adolescents (10.7%) than in Chinese adolescents (2.4%) [25, 28]. Few methodological differences possibly cause different recognition rates between studies (e.g., different vignettes and age of participants) but, it certainly implies the importance of improving Korean teenagers’ ability to recognize IGD and PSU.

### Perception towards a person with IGD and PSU

The current study also found that Korean teenagers generally acknowledged the difficulty of living with IGD and PSU, including difficulty being treated, less happiness, less successful academic life, and less satisfying friendship. However, they tended to have less empathy towards them. Empathy allows a person to connect and reach out with others and feel for them and plays a vital role in a person’s help-seeking attitudes. The previous study showed that empathy towards a person with mental illness was closely linked with help-seeking attitudes from professionals [40]. Therefore, an educational campaign needs to put an effort to improve teenagers’ empathy toward a person with IGD and PSU to enhance their help-seeking attitudes from professionals.

Moreover, there are several important differences in participants’ perceptions of a person living with IGD and PSU. Generally, participants considered that a case with IGD is more challenging to be treated and has a less successful academic life and less satisfying friendship. Such differences in their perception may stem from the fact that a higher number of teenagers in this study considered that the case with IGD has an addiction disorder. Therefore, participants perceived that the life of a person with IGD is more challenging than a person with PSU. This finding may suggest a strong like may exist between a person’s ability to recognize problems and perception of disorders.

### Help-seeking attitudes towards a person with IGD and PSU

Korean teenagers considered help from parents the most helpful for both vignettes, which was even rated higher than professional help. This finding is in line with the that of Lam (2017) who found that a higher percentage of Chinese adolescents preferred to seek help from mothers (24%) and fathers (16%), rather than seeking help from a school social worker or counselor (2%) [17]. Due to the limited number of MHL studies for IGD, none of the studies were carried out in western countries. However, there were previous MHL studies that investigated public’s MHL for other mental disorders in western countries and the finding showed that adolescents recommended help from a counselor (57.7%) for depression the most, followed by friends (41.8%) and family (40.8%) [41]. Therefore, a preference for seeking help from parents might be originated from an Asian culture where parents’ control is common in adolescents as parental control is a demonstration of love and interest in their child [42, 43]. On the other hand, when comparing participants’ help-seeking attitudes for a person with IGD and PSU, participants considered a person with IGD needs more professional help than a person with PSU. This difference could be generated from participants’ recognition of IGD as an addiction disorder and perception of the person with IGD as having a more challenging life. The result possibly shows that participants’ help-seeking attitudes are closely associated with the participant’s recognition and perception of disorders.

### Comparison of MHL depending on the low and high risk of IGD

The current study found that the high-risk group of IGD had a higher level of empathy towards a person with IGD than the low-risk group of IGD. Furthermore, although there were no significant group differences, the high-risk group of IGD was more likely to have positive perspectives on the outcomes of having IGD in terms of happiness, successful academic performance, and satisfying friendships. This result is in line with the previous findings that the level of IGD was positively associated with the positive outcome expectancy [44, 45]. Lin et al. (2008) examined the relationship between the positive or negative outcome expectancy and the level of Internet use in college students [44]. They found that students with a higher level of Internet addiction were more likely to have positive outcome expectancy [44]. Wu et al. (2016) also found a positive relationship between positive outcome expectancy of Internet gaming and IGD in adolescents [45]. These findings suggest that teenagers need to have appropriate perspectives on the outcome of having certain addiction disorders to prevent developing addiction disorders. The current study also found that the high-risk group of IGD perceived addiction counselors as the most helpful while the low-risk group rated the parents the most helpful. These results are likely to be due to participants’ past experience because it seems possible that some of the participants in the high-risk group may have experienced help from an addiction counselor since the Korean government put an effort to develop Internet addiction prevention centers to resolve the problem of overdependency on technology. South Korean government-funded National Information Society Agency launched the first Internet addiction prevention counseling center [46]. Since then, several projects have been developed, including prevention, counseling, and treatment [46]. A recent systematic review that examined the characteristics of intervention programs for Internet/smartphone addiction among adolescents from 2013 to 2017 found that 12 out of 14 studies were conducted in South Korea [47]. The most common intervention type was counseling (35.7%), followed by art therapy (21.5%), play therapy (14.3%), and integrated therapy (14.3%) [47]. Therefore, the high-risk group may have experienced support from addiction counselors and realized that addiction counselor is more helpful than parents.

### Comparison of MHL depending on the low and high risk of PSU

The current study also found that the high-risk group of PSU was more likely to recognize the correct label of PSU. The finding is consistent with previous research. The population-based government-funded project examined the Korean public’s smartphone overdependence in 2018 and found that compared to the general population, a high-risk group of PSU showed better recognition of problematic smartphone use both in themselves and in Korean society [48]. The high-risk group was more likely to perceive PSU as a problematic issue in Korean society and think they are experiencing PSU. However, despite the high awareness of the PSU in the high-risk group, their general help-seeking attitudes were significantly poorer than the low-risk group. This result contradicts findings from previous studies that recognition of disorders was associated with help-seeking attitudes from professionals [11, 12, 49]. For example, Picco et al. (2008) conducted population-based research to investigate an association between recognition and help-seeking preferences and found that the recognition of disorders was associated with an increased preference to seek help from professionals for dementia, depression, and schizophrenia [49]. There is a possibility that this inconsistency in findings might result from different methodologies employed in each study (e.g., vignettes depicted different disorders and different age ranges of participants). Therefore, further research on PSU is needed to confirm this association. However, there is no doubt about the need to improve help-seeking attitudes from professionals in the high-risk group of PSU.

The study has several limitations that need to be discussed. The primary limitation of this study is the use of the cross-sectional nature of the data. Therefore, the findings of the current study do not permit the causal link between the risk of IGD or PSU and the level of MHL. Second, the current study only recruited adolescents with a potential risk of addiction from the central city of South Korea. Therefore, this limit the generalizability of the findings to all adolescents in South Korea. Third, the study focused on adolescents’ MHL, particularly for IGD and PSU. As such, we cannot determine whether adolescents have poor MHL only for IGD and PSU, or Korean adolescents generally have poor MHL for other psychiatric disorders. Another limitation of the current study is that the study relied on adolescent self-reports when screening the risk of IGD and PSU. Since the IGD and PSU could be challenging behaviors that adolescents do not want to disclose, adolescents may not give honest responses. As a result, minimal differences in the level of MHL between low-risk and high-risk groups of addiction may be caused by reporting bias from self-reported measurements. Therefore, multiple informants (including parents) are needed when screening adolescents’ risk of IGD or PSU.

Despite these limitations, the current study certainly adds to our understanding of the adolescents’ ability to recognize IGD and PSU, and their perception and help-seeking attitudes towards the person with IGD and PSU. Prospective research is needed to conduct large community-based studies on adolescents’ MHL for behavioral addictions and other psychiatric disorders to confirm the findings in this study.

## Conclusions

To conclude, Korean teenagers had a poor ability to recognize IGD and PSU and were more prone to seek parental help than professional help. The current study also showed that teenagers had better recognition of IGD than PSU and perceived that a person with IGD has a more difficult life than a person with PSU. Furthermore, a low-risk or high-risk group of IGD or PSU showed minimal differences in MHL. Therefore, the current study provided empirical evidence to support the development of educational programs tailored to improve Korean teenagers’ MHL for both IGD and PSU. By doing so, future research on the effectiveness of educational programs to improve teenager’s MHL is recommended.

## Supporting information

S1 Dataset (SAV)
